# methylR: a graphical interface for comprehensive DNA methylation array data analysis

**DOI:** 10.1093/bioinformatics/btad184

**Published:** 2023-04-11

**Authors:** Massimiliano Volpe, Jyotirmoy Das

**Affiliations:** Bioinformatics Unit, Core Facility (KEF), Faculty of Medical and Health Sciences (BKV), Linköping University, Linköping SE-58185, Sweden; Clinical Genomics Linköping, SciLife Laboratory, Department of Biomedical and Clinical Sciences, Linköping University, Linköping, SE-58185, Sweden; Bioinformatics Unit, Core Facility (KEF), Faculty of Medical and Health Sciences (BKV), Linköping University, Linköping SE-58185, Sweden; Clinical Genomics Linköping, SciLife Laboratory, Department of Biomedical and Clinical Sciences, Linköping University, Linköping, SE-58185, Sweden

## Abstract

**Motivation:**

DNA methylation analysis using arrays is a widely used method in research and clinical studies to study Epigenetics. Although several packages have been published to incur the results, most of them require a deep computational knowledge to perform the analysis. To resolve the limitation and to offer an easily accessible solution for researchers, we developed *methylR* a graphical tool that can analyze not only the raw data but also performs different downstream analyses with a few mouse clicks.

**Results:**

We used standard and established open-source published packages or pipelines in *methylR*. We evaluated a publicly available dataset and compared the published results with those obtained with our tool. We implemented eight downstream analysis modules that can perform multidimensional analyses to pathway enrichment. Although the main application is designed for Illumina DNA methylation array data analysis, we made the accessory modules suitable for other kinds of data analysis as well.

**Availability and implementation:**

Freely available at Github: https://github.com/JD2112/methylr; Webserver: https://methylr.research.liu.se.

## 1 Introduction

DNA methylation is a hereditable epigenetic mechanism involving the transfer of a methyl group to cytosine at its 5-carbon position (CpG) mediated by the DNA methyltransferase enzyme family. Methylation occurring at cytosines in DNA represents the most stable type of epigenetic modification. Several studies show the role of DNA methylation in many different important biological processes (Yong et al. 2016) and in different diseases (Das et al. 2019, 2021). Methylation arrays, such as Illumina Infinium, are widely used due to lower cost and less processing time than sequencing. Besides Genome Studio, several open-source packages have been developed to analyze data from Illumina microarrays. *Minfi* (Aryee et al. 2014), *ChAMP* (Tian et al. 2017), *RnBeads* (Müller et al. 2019), and *lumi* (Du et al. 2008) are some packages available through Bioconductor that perform quality control, variance stabilization, normalization, and gene annotation for both Illumina 450K and HumanMethylationEPIC array. However, all these packages require advanced bioinformatics skills to be installed and used effectively.

To date, using DNA methylation data analysis through graphical user interface (GUI) is limited. Most of them are made in Shiny (Chang et al. 2021) an R library that allow to build efficient, functional, and graphically appealing interactive web apps which could then either run locally or hosted on a webserver.

We have found *ShinyÉPICo* (Morante-Palacios and Ballestar 2021) to be the most complete in this context, allowing users to perform filters, normalization, differentially methylated CpGs (DMCs), and differentially methylated regions calculation and export analysis results. However, it lacks functionalities to perform downstream analyses and visualize the results. Both *shinyMethyl* (Fortin et al. 2014) and *MethylAid* (van Iterson et al. 2014) offers a comprehensive shiny app for array design and visualization, quality control, and data normalization without any further calculation of DMCs while the former is also restricted to Illumina 450K array.

Here, we introduce *methylR*, a complete pipeline for the analysis of both 450K and EPIC Illumina arrays (850K) which aim to overcome all of these limitations by not only offering data visualization and normalization but also providing additional features such as the annotation of the genomic features resulting from the analysis, pairwise comparisons of DMCs with different graphical representation plus functional and pathway enrichment as downstream analyses. All these functionalities come packed in a minimal, elegant, and intuitive GUI which brings the analysis of DNA methylation array data within everyone’s reach. The *methylR* app comes packed as a container that could be run directly with one-line command and without any complex installation. Requiring low to no bioinformatics skills at all, *methylR*, represents a complete all-around easy tool to be adopted by scientists for Illumina DNA methylation array data analysis.

## 2 Methods

**Figure 1:**
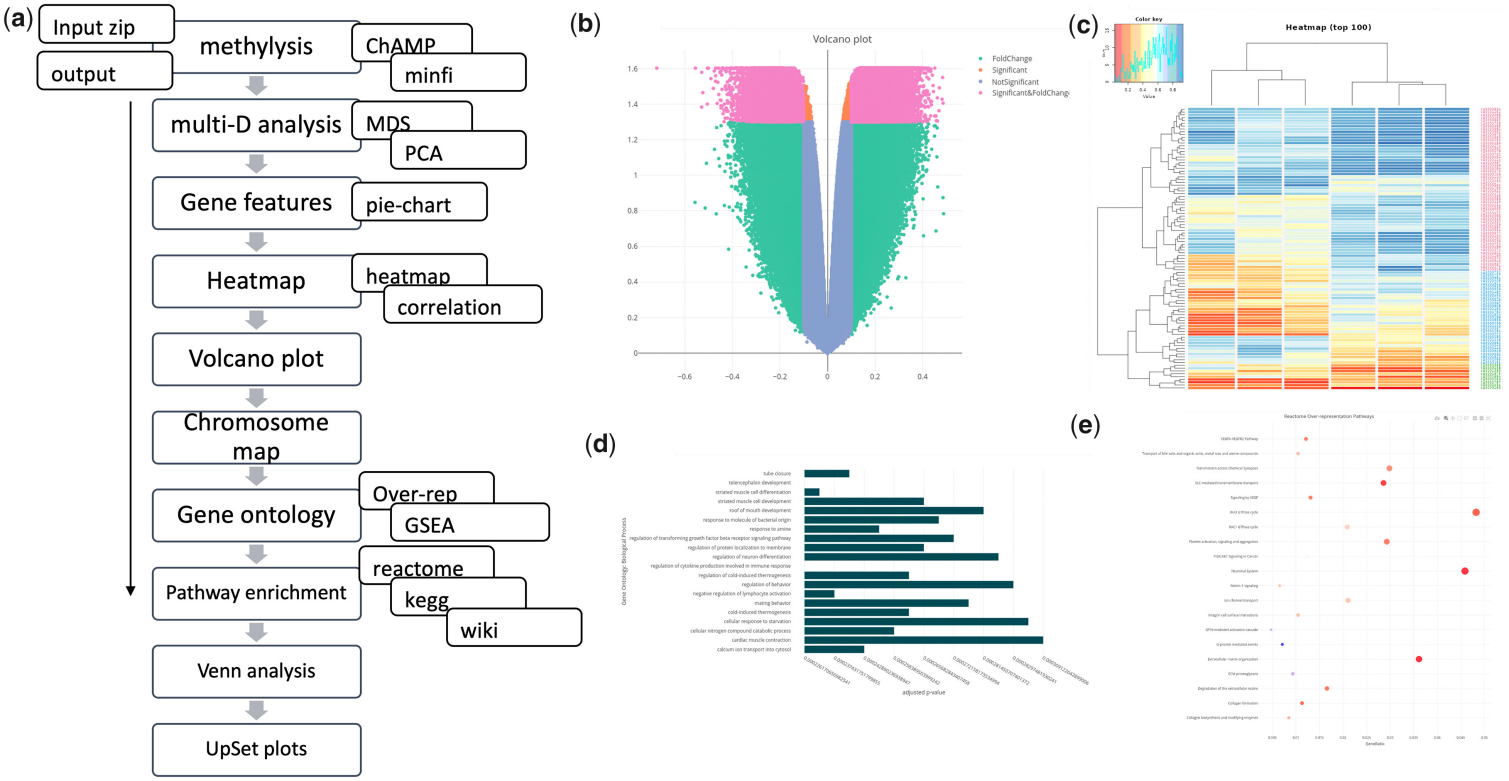
(a) Schematic diagram of the pipeline. (b) Volcano plot from using EPIC DNA methylation array. (c) Heatmap using top 100 CpGs normalized β values. (d) GO over-representation analysis result. (e) Pathway enrichment result using Reactome database and over representation analysis with BH-corrected *P*-value

The *methylR* dashboard is composed of different sections and it is designed to guide the user through the different steps of the analysis following a top to bottom organization. The first section is called *methylysis* and hosts the two main pipelines to perform the methylation arrays analysis, *ChAMP* (Tian et al. 2017) and *minfi* (Aryee et al. 2014). Both can compute the differential methylation analysis for EPIC or 450K Illumina arrays so it is up to the user to choose the algorithm to run, although we believe ChAMP better adapts to most of the user scenario and we suggest new users to start from that. Once the results of the main analysis with ChAMP (or alternatively minfi) are ready, they could be downloaded as they serve as input files for the downstream sections that will perform the exploration and representation of the results obtained (Figure 1a). Gene features, volcano, and enrichment plots are made by using *ggplot* (Wickham 2009) and *plotly* (Plotly Technologies Inc. 2015) R packages to provide clear and interactive graphical representation of the results. Figures can be downloaded as PNG, PDF, TIFF or SVG (static figures), or HTML (interactive) format. The latest full version of *methylR* is available on GitHub (https://github.com/JD2112/methylr) and can be run locally since it is packed in a container and could be run through a single-line command with either Docker (Merkel 2014) or Singularity (Kurtzer et al. 2017). We also setup a CentOS-based small webserver from the docker container that methylR user can run freely (https://methylr.research.liu.se). The performance may vary based on the user’s hardware availability; however, *methylR* is exceptionally low demanding in terms of resources. The *methylR* manual guides the user through the entire analysis to understand and tune the different important parameters for DNA methylation analysis. Besides this extensive manual, *methylR* comes with a support group (https://groups.google.com/g/methylr) to be in touch with the community. We successfully tested *methylR* on AMD64-based architecture in a Linux environment with 1 core and 12 GiB RAM and took approximately 3–4 min (*ChAMP*: 3 min, *minfi*: 4 min for the test data set) to run. We tested *methylR* with publicly available data coming from a cohort of patients affected by glioblastoma (GSE207426) and treated with radiotherapy or temozolomide, finding overlapping results (Lysiak et al. 2022). In particular, we found the same number of DMCs in the full dataset (123 510 DMCs) as well as in the TSS (both TSS200 and TSS1500) + 5′ UTRs subset (26 626 DMCs). We distribute a subset of the very same dataset through *SourceForge* (https://sourceforge.net/projects/methylr/) as a test dataset.

## 3 Application overview and steps

### 3.1 Methylysis section

This section allow the user to analyze the raw data with two independent pipelines, *ChAMP* or *minfi* with each of them having its separate subsection. *MethylR* allows to tune different parameters such as adjusted *P*-value, compute the batch effect correction or calculate cell type deconvolution, set filtration criteria, and select the annotation database (*minfi* only) to run the analysis. *MethylR* offers a large choice of methods to choose from to preprocess and normalize the raw data depending on the pipeline chosen and adds some unique features compared to ShinyÉPICo (see Supplementary Table S1). For example, the user can account for the batch effect and correcting for several factors. Once all parameters are set, the user can upload a zip file containing the raw data and the analysis will start automatically. At the end of the analysis, the box named “Methylation Analysis Result” will display the analysis results through different tabs and all the results can be downloaded as a zip. *MethylR* needs all the *idat* files (as they come from the Illumina instrument) with the csv file compressed in a zip archive. The section Appendix A (https://methylr.netlify.app/appendix1.html) of the manual covers how to make the zip file step by step. Once the analysis is done, the user can download the main results files. Those files could be used later to run the additional analyses modules offered by *methylR* as discussed in the next section.

### 3.2 Feature analysis section

#### 3.2.1 Multi-D analysis

Multiple dimensional (multi-D) analysis is offered either by MDS (Multidimensional Scaling) or PCA (Principal Component Analysis). The user can run both within the same module and compare the differences in the same subsection. Here, the number of variables and zoom levels can be selected while the plots generated can be exported in different image file formats.

#### 3.2.2 Gene features

By taking advantage of the annotation performed in the first step, this section provides the user with a summary of the genomic features intersecting the main results table. DMCs could either overlap Exons, UTRs, TSS, and this information is crucial since methylation at specific features, such as promoters, might alter the gene expression. This section comes with an interactive pie-chart illustrating the different percentages of the genomic features annotated. A comprehensive table of the annotation can be downloaded as a tab-delimited file.

#### 3.2.3 Pairwise and heatmap plots

Pairwise plot combines the correlation analysis and the interactive heatmap to visualize the CpG β/M-values. The input file can be uploaded in different format (matrix or list) or it could be generated by the user in the tab “matrix preparation.” The interactive heatmap with cluster analysis portrays the individual CpG methylation status (Figure 1c).

#### 3.2.4 Volcano plot section

Volcano plot is an essential visualization tool for differential methylation analysis. With the selection of adjusted *P*-value and logFC cutoff, an interactive volcano plot with significant list can be generated directly from the DMC data (Figure 1b).

#### 3.2.5 Chromosome plot section

Chromosome plot allows to map CpG against chromosomes to visualize the position of statistically significant DMCs over chromosome structure. As for the pairwise plot, the user can set cut-offs on both *P*-value and logFC values. It is possible to visualize one chromosome at a time or the full set.

### 3.3 Association study section

#### 3.3.1 Gene ontology (GO) analysis

Gene ontology (GO) analysis which is often required to understand the functions of the identified genes, can also be performed as a part of *methylR.* It offers an interactive plot selecting several parameters and a wide range of *P-*value adjustment methods. It also generates the data table for the user. Users can directly select the DMC file as input to generate the GO analysis result (Figure 1d).

#### 3.3.2 Pathway analysis

After the functional analysis, the biological pathways always come into the question and *methylR* offers the pathway analysis by using three different databases (KEGG, Reactome, and WikiPathways) for over-representation and gene-set enrichment analysis directly from the DMC list. It includes various *P*-value adjustment methods and selection of number of pathways and will generate a table of results in output (Figure 1e).

### 3.4 Set analysis

In this section, *methylR* allows to compare results from minimum two datasets analyses using Venn (2–6 datasets) or Upset (>6 datasets) plots. Results can be drawn from various settings and parameters mentioned in the section.

All the tools discussed in Sections 3.3 and 3.4 come with the possibility to be used standalone with results coming from other types of analyses (e.g. RNA-seq).

## 4 Conclusions

With the advancement of new sequencing technologies, DNA methylation array data analysis has become a routine practice. Due to the lack of user-friendly or subscription-free interfaces, it gets tiresome for scientists and here we designed a user-friendly freeware with GUI to reduce the effort. Our tool provides scientists with a free, quick, and easy all-in-one solution to perform a full DNA methylation array analysis from raw data to exploration and visualization of the results without the need of a background in bioinformatics. We plan to support and expand *methylR* and with continuous development, we expect to provide a complete and handy tool for DNA methylation data analysis.

## Supplementary Material

btad184_Supplementary_Data

## Data Availability

The fully-functional tool is publicly available on Docker container (https://hub.docker.com/r/jd21/methylr) and webserver (https://methylr.research.liu.se). The test data (or sample data) and manuals are publicly available on Github (https://github.com/JD2112/methylr), Sourceforge (https://sourceforge.net/projects/methylr/) and Netlify (http://methylr.netlify.app). The sample dataset was taken from a previously published data which is available on NCBI GEO database (https://www.ncbi.nlm.nih.gov/geo/query/acc.cgi?acc=GSE207426).
