# Supplementary material for: methylR: a graphical interface for comprehensive DNA methylation array data analysis
Source: Bioinformatics. 2023 Apr 11;39(4):btad184. doi: 10.1093/bioinformatics/btad184 (PMC10125902; doi:10.1093/bioinformatics/btad184)
Supplement: btad184_Supplementary_Data [file btad184_supplementary_data.pdf]

Supplementary Table 1: Feature comparison among different Shiny tools for DNA methylation data analysis

Tool name	Latest update	Deployment *	Array	Algorithms	QC	DMCs	DMRs	Heatmap	Chromosome	GO	Pathway	Set	Export results	PMID
ShinyÉPICo	2022	c; b	450k; 850k	minfi	yes	yes	yes	yes	no	no	no	no	yes	33416853
shinyMethyl	2017	b	450k	minfi	yes	no	no	no	no	no	no	no	no	25285208
MethylAid	2018	b	450k; 850k	minfi	yes	no	no	no	no	no	no	no	no	25147358
methylR	2022	c	450k; 850k	minfi; ChAMP	yes	yes	no	yes	yes	yes	yes	yes	yes	N/A

*b = bioconductor; c = container
